# Supplementary material for: Outcomes of kidney transplantation over a 16-year period in Korea: An analysis of the National Health Information Database
Source: PLoS One. 2021 Feb 19;16(2):e0247449. doi: 10.1371/journal.pone.0247449 (PMC7894945; doi:10.1371/journal.pone.0247449)
Supplement: Attachment — Submitted filename: Response to Reviewers.docx [file pone.0247449.s001.docx]

**Authors’ responses to the reviewers’ comments**

First of all, we greatly appreciate your favorable review of our manuscript. In addition, we would also like to thank you for your valuable comments and suggestions. In response to the points that you raised, we would like to offer the following answers.

**Reviewers’ comments**

The manuscript is the summary in the detail analysis of transplant outcomes in Korea during the last sixteen years. The study include data about: patient characteristics; immunosuppression therapy; incidence of graft rejection and graft loss. Although, it is a useful review about the kidney transplantation system in Korea, the authors need to address all specifically indicated aspects by the reviewers.

The authors need to show the type of donors. It is also necessary to clearly show the results with deceased donors vs. live donors. Cosequently, tables 1, 3 and 4 need to be modified to clearly show the results for deceased donors and for live donors. In addition, the appropiate statistical analysis needs to be performed. All these changes will require significant revisions to the manuscript.

**Response:** We greatly appreciate your valuable opinions on our manuscript. We have added the results on the analysis of donor types and have revised Tables 1, 3, 4, and 5. We also have added descriptions regarding results of additional analyses to the revised manuscript page 5, 7, 11, 12, 14, and 17. We also have revised Fig 2 and 3.

The authors need to provide enough information supported by the appropiate statistical analysis to make sure that there is not an over-interpratation of their results.

The authors need to address specific comments by the second reviewer: ABO compatibility (whether or not ABOi transplants were included), and pre-transplant sensitization levels.

The conclusions need to be re-evaluated regarding risk factors impacting transplantation outcomes in Korea.

Very careful ststistical analysis for graft failure risk needs to be performed using a Cox regression model, testing for proportional hazard distribution with variables.

**Response:** We agree with the opinion on the necessity of analyzing clinical data including ABOi and pre-transplant sensitization level. Unfortunately, it is difficult to investigate detailed clinical information, because we analyzed data from the database of the National Health Insurance claims. We have added some descriptions as the limitations of our study, and also have revised conclusions of our study (page 20-21) to reflect the reviewer’s suggestion. We also have reanalyzed the Cox’s regression by adding the donor types as a variable (Table 5). Cox regression model showed that the recent KT (during 2010-2017) have 9.732 hazard ratio when compared to preceding period (during 2002-2009). We also have added Fig 3B which reflects grafts survival according to the periods of KT to check and visualize the results of survival analyses.

**In response to the reviewer # 1:**

The presented data are not robust, and not in-depth. Additional data need to be included divided into deceased and living donors. The results need to reflect the standard reporting of transplant data accepted universally for example 'survival free rejection' is no commonly used. Overall survival vs graft survival, % acute rejection (report Tcell vs AMR vs Mixed), what % was deceased donor vs Living Donor kidney tx., HLA match, ABO vs ABOi, Desensitization protocol used, ATG dosing?. It is particularly interesting and counter to accepted results that ATG use led to worse outcomes. The authors do not explain this finding. The thesis was mixed, a more descriptive manuscript would suffice with the data and findings clearly presented.

**Response:** We have added the results on the analysis of donor types and have revised Tables 1, 3, 4, and 5, and the results of additional analyses have been added to the revised manuscript (page 5, 7, 11, 12, 14, and 17). We also have revised Fig 2 and 3. We agree with the reviewer’s opinion on the necessity of analyzing detailed clinical data including HLA match, ABO vs ABOi, desensitization protocol used. Because we analyzed data from the database of the National Health Insurance claims, it is difficult to investigate detailed clinical information and we have described these as the limitations of our study (page 20-21). In the current study, the use of ATG was analyzed as a variable which can be associated with for graft failure. Several recent studies have reported that the ATG induction show superiority over basiliximab induction for the prevention of acute rejection after KT. However, since the current study is a retrospective cohort study, it is not plausible to interpret that the use of ATG is direct cause of graft failure. It seems appropriate to interpret our results as a temporal relationship. We have added some descriptions on the interpretation of our data to the discussion section (page 19).

**In response to Reviewer # 2:**
This neatly organized and well written study summarizes the analyses of transplant outcomes in Korea over the last sixteen years. The manuscript is a useful presentation of transplantation experience in this country. Results on patient characteristics, immunosuppression therapy use and as wells graft rejection and loss statistics are presented. Overall, it is a good manuscript for readers wishing to learn about kidney transplantation system in Korea. Some important details were missed, however. Most importantly, please kindly indicate the donor type in the patient cohort. Were all patients included in the study a deceased donor transplant recipients, or it was a mix of deceased and living donor transplant recipients. If the latter is the case, donor type (and donor relationship for living donors) should be shown in Tables 1, 3 and 4, and, importantly, included in Cox regression model as a variable and shown in Table 5. This would constitute a major revision to the manuscript.

**Response:** We greatly appreciate your valuable opinions on our manuscript. We have added the results on the analysis of donor types and have revised Tables 1, 3, 4, and 5. We also have added descriptions regarding results of additional analyses to the revised manuscript page 5, 7, 11, 12, 14, and 17. We also have revised Fig 2 and 3.

Overall, I would caution against over-interpreting the results of variable distribution comparison between patients with or without acute rejection and graft loss, and Cox regression, due to the lack of information on donor type, ABO compatibility (whether or not ABOi transplants were included), pre-transplant sensitization level. The conclusions of the paper regarding risk factors impacting transplantation outcomes in Korea should be at least paraphrased to reflect these limitations. In addition, for a truly vigorous statistical testing of graft failure risk in a Cox regression model, testing for proportional hazard distribution of included variables would be beneficial, though I consider it optional for the scope of this manuscript.

**Response:** We agree with the reviewer’s opinion on the necessity of analyzing clinical data including ABOi and pre-transplant sensitization level. Unfortunately, it is difficult to investigate detailed clinical information, because we analyzed data from the database of the National Health Insurance claims. We have added some descriptions as the limitations of our study, and also have revised conclusions of our study (page 20-21) to reflect the reviewer’s suggestion. We also have reanalyzed the Cox’s regression by adding the donor types as a variable (Table 5). Cox regression model showed that the recent KT (during 2010-2017) have 9.732 hazard ratio when compared to preceding period (during 2002-2009). We also have added Fig 3B which reflects grafts survival according to the periods of KT to check and visualize the results of survival analyses.
